# Cumulative exposure to shift work and sickness absence: associations in a five-year historic cohort

**DOI:** 10.1186/s12889-016-3906-z

**Published:** 2017-01-11

**Authors:** Alwin van Drongelen, Cécile R.L. Boot, Hynek Hlobil, Allard J. van der Beek, Tjabe Smid

**Affiliations:** 1 Department of Public and Occupational Health, EMGO Institute for Health and Care Research, VU University Medical Center, PO Box 7057, Amsterdam, 1007 MB The Netherlands; 2 KLM Health Services, Schiphol Airport, Schiphol, The Netherlands; 3 Body@Work TNO VUmc, Research Center on Physical Activity, Work and Health, VU University Medical Center, Amsterdam, The Netherlands

**Keywords:** Work schedule tolerance, Sick leave, Cohort studies

## Abstract

**Background:**

Exposure to shift work has been associated with negative health consequences, although the association between shift work and sickness absence remains unclear. The aim of this study is to investigate associations between cumulative exposure to shift work and sickness absence among ground staff employees of an airline company.

**Methods:**

This study used data from the MORE (Monitoring Occupational Health Risks in Employees) cohort, which is a 5-year historic cohort. The population of the present study consisted of 7562 ground staff employees. For each employee, work schedules and sickness absence days between 2005 and 2009 were obtained from company records. For the exposure to different shift schedule types and to the cumulative number of night shifts, the association with long-term sickness absence (>7 consecutive sickness absence days) and the number of sickness absence episodes during 2009, was calculated using logistic and Poisson regression analyses. Socio-demographic variables, work-related variables, job classification variables, and previous sickness absence days were regarded as confounders.

**Results:**

After adjusting for previous sickness absence and job classification variables, only the group of employees that switched into working in a three-shift schedule, showed a significantly increased risk for long-term sickness absence (OR = 1.31, 95%CI 1.02–1.69). Night shift exposure was not significantly associated with long-term sickness absence. Exposure to shift work was negatively associated with more sickness absence episodes. Employees who were exposed to more than 46 night shifts also showed a lower risk for more sickness absence episodes. Subgroup analyses showed that single employees and employees without children had an increased risk for long-term sickness absence when exposed to a three-shift schedule, and when they had changed between shift schedule types.

**Conclusions:**

Cumulative exposure to shift work proved to be negatively associated with more sickness absence episodes, and was not associated with more long-term sickness absence, although selection bias could not be ruled out. Future research should explore the influence of household composition, and take into account both previous sickness absence and psychosocial and physical work factors to obtain a better estimation of the association between shift work and sickness absence.

## Background

The current 24-h economy makes it necessary for employees to work outside the once regular 9 to 5 working hours, in the early morning, evening, and night. Consequently, it has been estimated that almost 20% of the work force in Europe and the USA works in shift work [1, 2]. Exposure to shift work has become an important topic in occupational health as it has been associated with negative consequences for the employee. Short-term effects comprise disturbed sleep, increased fatigue [3], and work-family conflict [4]. Long-term risks involve cardiovascular disease, gastro-intestinal problems [5–9], metabolic disturbances [10–12] and cancer [13, 14].

Sickness absence is considered to be a risk factor for health deterioration and mortality [15, 16], and can have unfavourable financial consequences for the individual, employer and society [17, 18]. Work-related risk factors for sickness absence involve both physical (e.g. ergonomic factors, work schedules) and psychosocial [e.g. job strain, social support] characteristics [19–22]. In the literature, a distinction is made between short- and long-term sickness absence [21, 23]. Long-term sickness absence contributes disproportionally to the total sickness absence costs while it accounts for a small fraction of the number of absence episodes. For the employees, long-term sickness absence can be associated with future sickness absence, an increased risk for work disability, and both social and financial problems [17, 18, 23, 24]. Short-term sickness absence, on the other hand, can be part of a behavioural pattern, or of a coping strategy to prevent long-term sickness absence [20, 25].

To obtain a better understanding of the consequences of shift work, it is necessary to investigate the association between exposure to shift work and both short- and long-term sickness absence. Although shift work in relation to sickness absence has been studied before, its association remains unclear [26–28]. Merkus et al. [29] stated that sickness absence should be studied per specific population and shift work schedule. However, it is also possible that it is not the specific schedule, but the cumulative exposure to night shifts that imposes health effects, due to the chronic disruption of the circadian rhythm [30–32].

Therefore, this study aims to investigate the associations between cumulative exposure to different types of shift schedules and cumulative exposure to night shifts on the one hand, and the number of sickness absence episodes and long-term sickness absence on the other, among ground staff employees of a large international airline company.

## Methods

### Sources of information and study population

This study used data from the MORE (Monitoring Occupational Health Risks in Employees) cohort. This 5-year historic cohort was set up to analyze occupational health risks of employees of a large airline company and consists of all workers who were employed at the company at January 1, 2010. The cohort data comprised of sickness absence and human resource records of the employees of the airline company, since January 1, 2005. The human resource records included socio-demographic and work-related information, and individual work schedules that were registered for each day of the cohort period. The datasets were combined, anonymized, and coded by an independent occupational physician. According to Dutch law, this study was exempt from Medical Ethical review.

**Fig. 1:**
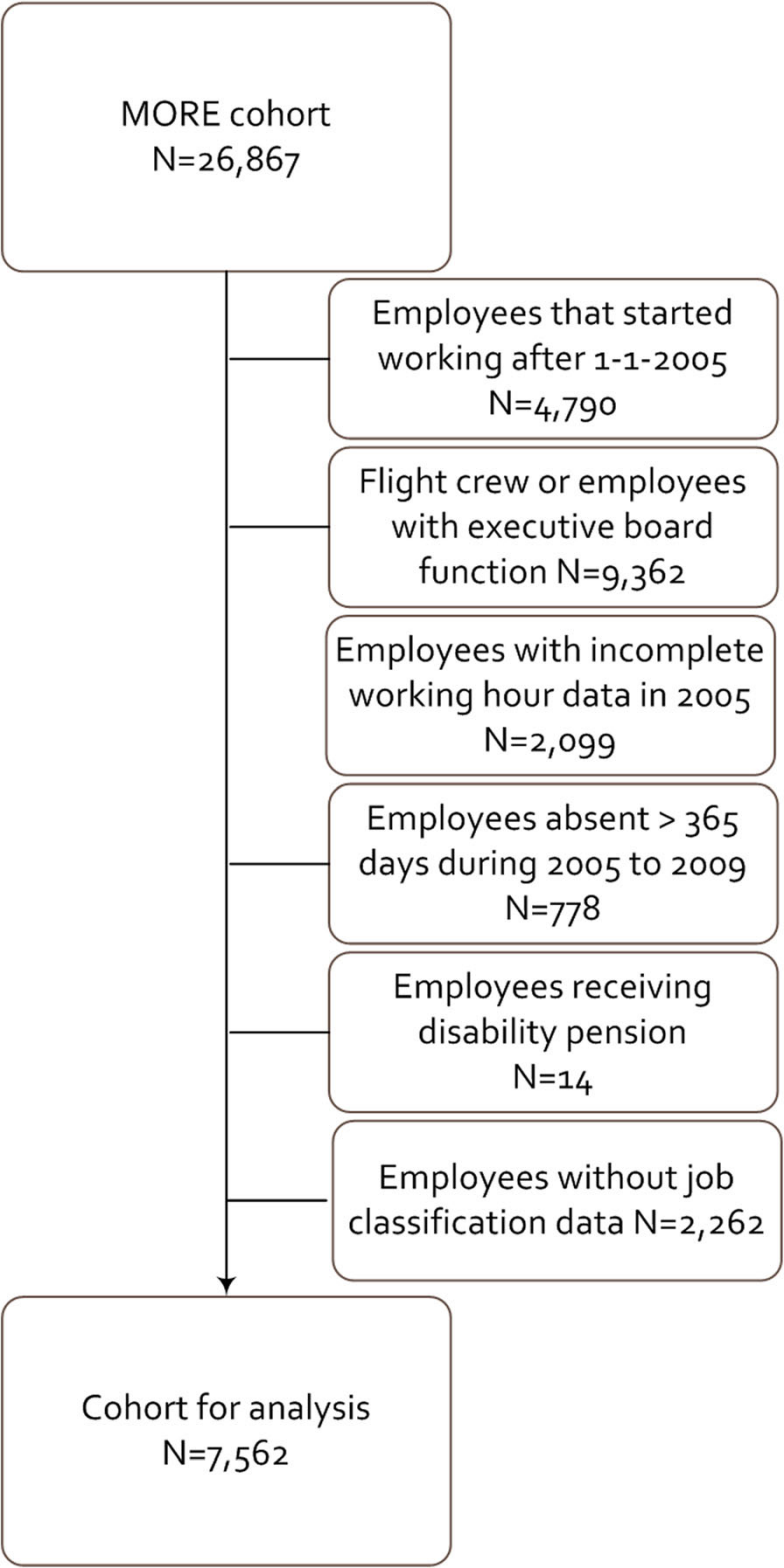
Flow chart of the exclusion procedure

The study population consisted of ground staff employees of the airline company that were already employed at January 1, 2005, and had complete working hour data during 2005. Employees were excluded if they received a disability pension at January 1, 2005, or had more than 365 days of cumulative sickness absence during 2005 to 2008. Furthermore, only employees with complete data on job classification variables were included. In total, 7652 employees were available for the analyses (Fig. 1).

### Outcome

Sickness absence was defined as registered absence from work due to health reasons. All employees of the company contacted the occupational health service when they called in sick, and when they wanted to return to work. The occupational health service digitally registered all sickness absence records. For this study, calendar days of the registered sickness absence episodes were counted as sickness absence days. Partial absence days were considered as full days of sickness absence [21].

The outcome measures were calculated for the period of January 1, 2009 until January 1, 2010.

1. First, the number of sickness absence episodes was calculated for each employee. Each episode was considered to be finished as soon as the employee fully returned to work, for at least 1 day [33].
2. Next, a dichotomous outcome variable was created to analyse long-term sickness absence in 2009. This variable was defined as at least one episode of more than seven consecutive sickness absence days. Previously, this cut-off has been used to determine medically certified sickness absence, for instance in the Whitehall II cohort [16, 34].

### Exposure

Daily work schedules were analyses for the period 2005 to 2008. For each eligible employee, the number of night shifts (shifts that comprised more than one hour between 0:00 and 6:00) was counted, and categorized into tertiles. Although it has been recommended to assess shift work exposure as extensive as possible [35], the shift work schedules of our study population showed a wide variation in rotation, speed and direction. Therefore, the following basic categories were created:

- Day work (no shifts)
- Three-shift schedule (rotating between morning/day, evening, and night shifts)
- Two-shift schedule (rotating between morning/day and evening shifts)

**Fig. 2:**
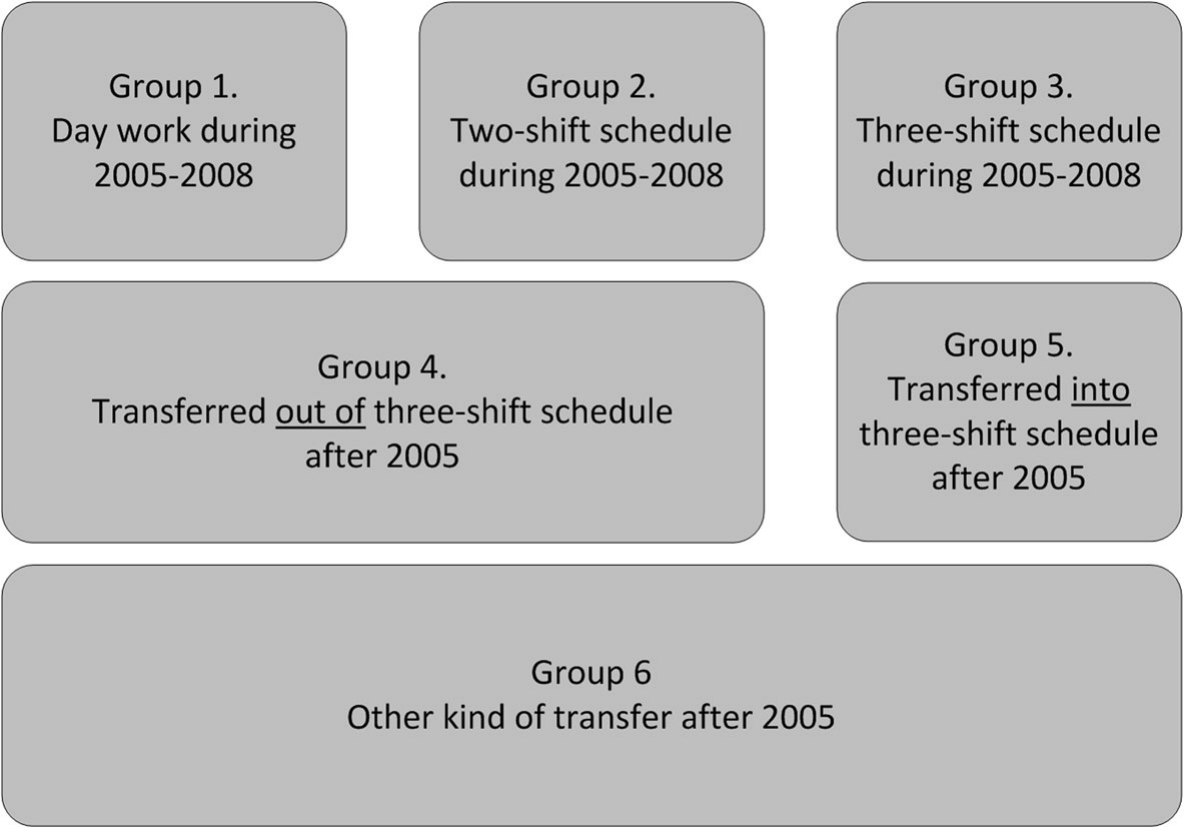
Composition of the different shift type groups

Subsequently, it was determined in which of these shift schedules the employee had worked during each year of the period 2005 to 2008. With this information, the employees were divided into six different groups, depending on the shift schedule exposure during 2005 to 2008 (Fig. 2).

### Other variables

Based on scientific literature, the following available variables were regarded as potential confounders and retrieved from the database at January 1, 2009 [19–23]. If the exact value was not available at that date, the proxy at January 1, 2010 was used. Skewed variables were divided into quartiles.

Included socio-demographic variables were age, gender, marital status (married/cohabiting, single, divorced/widow), having children (no, yes), and commuting distance (0 up to 16, 16 up to 24, 24 up to 41, >41 km).

Work-related variables were employment years, mean contract percentage during 2009 (0 up to 50%, 51 up to 80%, 81 up to 99%, and 100%), and working overtime during 2005–2008 (no, yes).

By means of the job classification system that was used within the company, all job titles (*n* = 1171) were systematically scored by independent evaluators, on a continuous scale on the following characteristics: responsibility (potential problems and corresponding effect), knowledge requirements (knowledge level), social interaction (leadership, expression, contact), special operational requirements (movement skills, degree of attention, needed exceptional qualities), and aggravated working conditions (physical exercise, working conditions, the level of personal risk) [36]. The job classification system was developed by the largest Dutch Employers Organization and is widely used throughout the country. Due to multicollinearity, only the scores on knowledge requirements (necessary specific knowledge, range 0–90), special operational requirements (required skills, range 0–18), and aggravated working conditions (physiological and physical workload, range 0–22) were used as potential confounders. The variable aggravated working conditions was skewed and therefore divided into quartiles. Finally, the total number of sickness absence days during 2005 to 2008 was calculated, and divided into quartiles (0 up to 26 days, 26 up to 67 days, 67 up to 140 days, ≥ 140 days). This will be referred to as previous sickness absence.

#### Analyses

Means, standard deviations and frequencies were calculated for the socio-demographic variables, the work schedule variables, the number of sickness absence days and episodes, and long-term sickness absence episodes (>7 days).

The associations of different shift schedule types and cumulative night shift exposure with long-term sickness absence (yes/no) were calculated by performing logistic regression analyses. The associations of the shift schedule types and cumulative night shift exposure with the number of sickness absence episodes were determined using Poisson regression analysis, correcting for overdispersion [37].

For each analysis, three models were composed. First, the crude association was determined. Next, the socio-demographic variables, work-related variables, and previous sickness absence days were added to construct the semi-adjusted model. A pre-selection procedure indicated that the selected potential confounders were univariately (*p* < 0.1) associated with the outcome measure. Finally, the job classification variables were added to create the fully adjusted models.

Interaction terms with age, gender, having children, and marital status were added to the crude model to detect possible effect modification. If the interaction term was statistically significant, subgroup analyses were performed.

A two-tailed significance level of *p* < 0.05 was considered to be statistically significant. Statistical analyses were conducted with the Statistical Package for Social Sciences (SPSS) version 20.0.

## Results

The socio-demographic, work-related and sickness absence characteristics of the study population are presented in Table 1. The sample consisted of 7562 employees with an average age of 45.4 years. The majority of the population was male (81.6%), had a fulltime contract (73.9%), and worked in a three-shift schedule during 2005 to 2008 (42.7%). In 2009, on average, the employees reported sick for more than 26 days, with a mean frequency of 1.7 episodes. For 48.6% of the employees at least one sickness absence episode of more than 7 days was registered.

**Table 1:** Socio-demographic, work schedule variables, and sickness absence characteristics

Characteristic		Number	Percentage
Age, mean (SD) [range]		7562	45.4 (8.0) [22–63]
Gender	Male	6167	81.6
	Female	1395	18.4
Marital status	Married/cohabiting	5667	74.9
	Single	1355	17.9
	Divorced/Widow	540	7.1
Children	No	2121	28.0
	Yes	5441	72.0
Employment duration (in months), mean (SD) [range]		7562	18.9 (9.2) [4–45]
Contract rate 2009	0 to 50%	299	4.0
	51 to 80%	1178	15.6
	81 to 99%	498	6.6
	100%	5587	73.9
Working overtime	No	2266	30.0
	Yes	5296	70.0
Knowledge requirements, mean (SD) [range]		7562	34.6 (13.0) [0–90]
Special operational requirements, mean (SD) [range]		7562	9.6 (4.0) [0–18]
Aggravated working conditions, mean (SD) [range]		7562	9.4 (6.0) [0–22]
Shift type group	Day work	1060	14.0
	Three-shift schedule	3232	42.7
	Two-shift schedule	1615	21.4
	Transfer out of three-shift	425	5.6
	Transfer into three-shift	595	7.9
	Other kind of transfer	635	8.4
Number of night shifts 2005–2008	No night shifts	2837	37.5
	Night shifts (1 to ≤46)	1589	21.0
	Night shifts (47 ≤ 110)	1572	20.8
	Night shifts (>110)	1564	20.7
Number of sickness absence days in 2009, mean (SD) [range]		7562	26.6 (45.5) [0–365]
Number of sickness absence episodes in 2009, mean (SD) [range]		7562	1.7 (1.5) [0–13]
Long-term sickness absence (>7 days)	No	3886	51.4
	Yes	3676	48.6

The associations between shift schedule exposure and sickness absence are presented in Table 2. In the crude and semi-adjusted models, all shift schedule groups, except for the group that transferred out of the three-shift schedule, showed an increased risk of long-term sickness absence compared to day workers. After adding the job classification variables to the model, only the group of employees that transferred into a three-shift schedule showed an increased risk for long-term sickness absence (OR = 1.31, 95%CI 1.02–1.69). In both the semi-adjusted and the fully adjusted model, all shift schedule types had a lower incidence risk ratio (IRR) for the number of sickness absence episodes, compared to the group that worked during the day.

**Table 2:** Crude, semi-adjusted, and fully adjusted models for shift schedule types and sickness absence outcomes

		Crude model	Semi-adjusted model	Fully adjusted model
Long-term sickness absence (>7 days)		OR (95% CI)	OR (95% CI)	OR (95% CI)
Shift schedule type 2005–2008	Day work	Reference	Reference	Reference
	Three-shift schedule	**1.88 (1.63–2.17)**	**1.43 (1.22–1.69)**	1.13 (0.94–1.35)
	Two-shift schedule	**1.76 (1.50–2.06)**	**1.40 (1.17–1.68)**	1.08 (0.89–1.31)
	Transfer out of three-shift	1.23 (0.97–1.54)	1.06 (0.82–1.37)	0.90 (0.69–1.17)
	Transfer into three-shift	**2.49 (2.03–3.06)**	**1.84 (1.46–2.32)**	**1.31 (1.02–1.69)**
	Other kind of transfer	**1.59 (1.30–1.94)**	**1.33 (1.06–1.67)**	1.06 (0.84–1.35)
Sickness absence days 2005–2008	≥0 to <26 days		Reference	Reference
	≥26 to <67 days		3.06 (2.58–3.62)	2.99 (2.52–3.55)
	≥67 to <140 days		8.10 (6.80–9.66)	7.45 (6.23–8.90)
	≥140 days		12.53 (10.48–14.99)	11.28 (9.40–13.53)
Special operational requirements				1.00 (0.99–1.02)
Knowledge requirements				0.98 (0.97–0.98)
Aggravated working conditions	≤4			Reference
	>4 to ≤11			1.14 (0.94–1.37)
	>11 to ≤15			1.14 (0.92–1.40)
	>15			1.21 (0.94–1.55)
Sickness absence episodes		IRR (95% CI)	IRR (95% CI)	IRR (95% CI)
Shift schedule type 2005–2008	Day work	Reference	Reference	Reference
	Three-shift schedule	0.95 (0.89–1.01)	**0.82 (0.77–0.87)**	**0.82 (0.76–0.88)**
	Two-shift schedule	0.95 (0.89–1.02)	**0.84 (0.78–0.90)**	**0.83 (0.77–0.90)**
	Transfer out of three-shift	0.93 (0.84–1.02)	**0.83 (0.75–0.91)**	**0.83 (0.75–0.92)**
	Transfer into three-shift	1.05 (0.96–1.14)	**0.87 (0.79–0.94)**	**0.89 (0.81–0.98)**
	Other kind of transfer	0.97 (0.89–1.06)	**0.82 (0.75–0.89)**	**0.83 (0.76–0.91)**
Sickness Absence days 2005–2008	≥0 to <26 days		Reference	Reference
	≥26 to <67 days		2.20 (2.06–2.35)	2.22 (2.08–2.37)
	≥67 to <140 days		3.18 (2.97–3.40)	3.22 (3.01–3.44)
	≥140 days		3.51 (3.28–3.75)	3.57 (3.34–3.82)
Special operational requirements				1.01 (1.00–1.02)
Knowledge requirements				1.00 (1.00–1.00)
Aggravated working conditions	≤4			Reference
	>4 to ≤11			1.03 (0.96–1.10)
	>11 to ≤15			1.03 (0.95–1.12)
	>15			0.86 (0.78–0.94)

Semi-adjusted model adjusted for age, gender, marital status, having children, commuting distance, employment duration, mean contract percentage, working overtime, and previous sickness absence days. Job classification variables special operational requirements, knowledge requirements, and aggravated working conditions were added to the fully adjusted model

Bold: *P* < 0.05

The associations between cumulative night shift exposure and sickness absence are presented in Table 3. In the crude and semi-adjusted models, employees with moderate night shift exposure (up to 110 night shifts between 2005 and 2008) were positively associated with more long-term sickness absence in 2009, compared to employees who had not been exposed to night shifts. After adding the job classification variables, night shift exposure was no longer significantly associated with long-term sickness absence. In the semi-adjusted model, all employees with night shift exposure showed a significant lower IRR for the number of sickness absence episodes compared to employees who had not been exposed to night shifts. In the fully adjusted model this was only the case for the employees who were exposed to more than 46 night shifts.

**Table 3:** Crude, semi-adjusted, and fully adjusted models for night shift exposure and sickness absence outcomes

		Crude model	Semi-adjusted model	Fully adjusted model
Long-term sickness absence (>7 days)		OR (95% CI)	OR (95% CI)	OR (95% CI)
Number of night shifts 2005–2008	No night shifts	Reference	Reference	Reference
	Night shifts (1 to ≤46)	**1.36 (1.20–1.53)**	**1.30 (1.12–1.50)**	1.15 (0.99–1.34)
	Night shifts (47 ≤ 110)	**1.54 (1.36–1.74)**	**1.17 (1.01–1.34)**	1.01 (0.87–1.17)
	Night shifts (>110)	1.08 (0.95–1.22)	1.06 (0.92–1.21)	1.05 (0.91–1.21)
Sickness absence days 2005–2008	≥0 to <26 days		Reference	Reference
	≥26 to <67 days		3.08 (2.60–3.65)	2.99 (2.52–3.55)
	≥67 to <140 days		8.11 (6.81–9.66)	7.42 (6.21–8.86)
	≥140 days		12.56 (10.50–15.02)	11.23 (9.36–13.48)
Special operational requirements				1.00 (0.98–1.02)
Knowledge requirements				0.98 (0.97–0.98)
Aggravated working conditions	≤4			Reference
	>4 to ≤11			1.16 (0.97–1.39)
	>11 to ≤15			1.18 (0.96–1.45)
	>15			1.26 (0.98–1.61)
Sickness absence episodes		IRR (95% CI)	IRR (95% CI)	IRR (95% CI)
Number of night shifts 2005–2008	No night shifts	Reference	Reference	Reference
	Night shifts (1 to ≤46)	1.03 (0.97–1.08)	**0.93 (0.88–0.98)**	0.97 (0.91–1.02)
	Night shifts (47 ≤ 110)	0.99 (0.94–1.04)	**0.87 (0.83–0.92)**	**0.91 (0.86–0.96)**
	Night shifts (>110)	**0.94 (0.89–0.99)**	**0.95 (0.90–1.00)**	**0.93 (0.88–0.98)**
Sickness absence days 2005–2008	≥0 to <26 days		Reference	Reference
	≥26 to <67 days		2.19 (2.05–2.34)	2.21 (2.06–2.35)
	≥67 to <140 days		3.16 (2.95–3.38)	3.20 (2.99–3.43)
	≥140 days		3.49 (3.27–3.73)	3.56 (3.32–3.81)
Special operational requirements				1.01 (1.00–1.02)
Knowledge requirements				1.00 (0.99–1.00)
Aggravated working conditions	≤4			Reference
	>4 to ≤11			1.03 (0.96–1.11)
	>11 to ≤15			1.04 (0.96–1.13)
	>15			0.87 (0.79–0.96)

Semi-adjusted model adjusted for age, gender, marital status, having children, commuting distance, employment duration, mean contract percentage, working overtime, and previous sickness absence days. Job classification variables special operational requirements, knowledge requirements, and aggravated working conditions were added to the fully adjusted model

Bold: *P* < 0.05

The interaction terms having children (*p* = 0.005) and marital status (*p* = 0.027) had a significant effect on the association between shift type and long-term sickness absence (crude model). Otherwise, none of the added interaction terms had a significant effect. Table 4 presents the results of the subgroup analyses. Employees without children showed to have a higher risk for long-term sickness absence in the fully adjusted model when they worked in a three-shift schedule, transferred into a three-shift schedule, or made another kind of shift schedule transfer. Comparing the three marital status groups, the fully adjusted model showed that married/cohabiting employees in the different shift schedule types did not have a significantly higher risk for long-term sickness absence compared to married/cohabiting day workers. In all models, single employees who worked in a three-shift schedule or made a shift schedule transfer did show significantly higher ORs for long-term sickness absence compared to single day workers. Divorced or widowed employees that transferred into a three-shift schedule after 2005 had an increased risk for long-term sickness absence compared to divorced or widowed day workers.

**Table 4:** Crude, semi-adjusted. and fully adjusted models for the subgroup analyses of shift schedule types and long-term sickness absence

		Crude model	Semi-adjusted model	Fully adjusted model
		OR (95% CI)	OR (95% CI)	OR (95% CI)
Without children (*n* = 2121)				
Shifttype 2005–2008	Day work	Reference	Reference	Reference
	Three-shift schedule	**2.14 (1.61–2.85)**	**1.80 (1.30–2.50)**	1.40 (0.97–2.01)
	Two-shift schedule	**2.55 (1.86–3.51)**	**2.34 (1.62–3.36)**	**1.76 (1.19–2.61)**
	Transfer out of three-shift	1.22 (0.75–1.99)	1.03 (0.60–1.75)	0.89 (0.52–1.55)
	Transfer into three-shift	**3.06 (2.07–4.52)**	**2.43 (1.55–3.81)**	**1.76 (1.08–2.87)**
	Other kind of transfer	**2.58 (1.80–3.71)**	**2.16 (1.41–3.28)**	**1.74 (1.12–2.69)**
With children (*n* = 5441)				
Shifttype 2005–2008	Day work	Reference	Reference	Reference
	Three-shift schedule	**1.79 (1.52–2.12)**	**1.34 (1.10–1.62)**	1.05 (0.85–1.30)
	Two-shift schedule	**1.53 (1.27–1.84)**	1.18 (0.96–1.46)	0.91 (0.73–1.14)
	Transfer out of three-shift	1.19 (0.92–1.55)	1.09 (0.81–1.46)	0.90 (0.67–1.22)
	Transfer into three-shift	**2.34 (1.83–2.98)**	**1.68 (1.28–2.21)**	1.19 (0.88–1.60)
	Other kind of transfer	**1.31 (1.03–1.67)**	1.09 (0.83–1.43)	0.85 (0.63–1.13)
Married/cohabiting (*n* = 5667)				
Shifttype 2005–2008	Day work	Reference	Reference	Reference
	Three-shift schedule	**1.84 (1.56–2.17)**	**1.38 (1.14–1.68)**	1.10 (0.89–1.35)
	Two-shift schedule	**1.60 (1.33–1.92)**	**1.27 (1.03–1.57)**	0.99 (0.79–1.24)
	Transfer out of three-shift	1.17 (0.90–1.53)	1.04 (0.78–1.39)	0.88 (0.65–1.18)
	Transfer into three-shift	**2.27 (1.79–2.89)**	**1.67 (1.28–2.19)**	1.18 (0.88–1.58)
	Other kind of transfer	**1.38 (1.09–1.75)**	1.17 (0.89–1.53)	0.93 (0.70–1.23)
Single (*n* = 1355)				
Shifttype 2005–2008	Day work	Reference	Reference	Reference
	Three-shift schedule	**2.23 (1.56–3.19)**	**1.90 (1.26–2.89)**	1.36 (0.86–2.17)
	Two-shift schedule	**3.18 (2.15–4.72)**	**2.66 (1.67–4.23)**	**1.82 (1.10–3.01)**
	Transfer out of three-shift	1.64 (0.90–3.00)	1.22 (0.62–2.39)	1.03 (0.51–2.05)
	Transfer into three-shift	**3.23 (2.00–5.21)**	**2.45 (1.41–4.27)**	1.66 (0.90–3.03)
	Other kind of transfer	**2.75 (1.75–4.32)**	**2.29 (1.35–3.90)**	**1.84 (1.06–3.18)**
Divorced/widow (*n* = 540)				
Shifttype 2005–2008	Day work	Reference	Reference	Reference
	Three-shift schedule	**1.73 (1.04–2.86)**	1.43 (0.80–2.57)	1.20 (0.63–2.27)
	Two-shift schedule	1.25 (0.73–2.14)	1.21 (0.66–2.23)	1.00 (0.52–1.96)
	Transfer out of three-shift	0.97 (0.42–2.28)	1.01 (0.38–2.66)	0.89 (0.32–2.45)
	Transfer into three-shift	**5.97 (2.23–15.97)**	**4.90 (1.61–14.94)**	**3.96 (1.24–12.69)**
	Other kind of transfer	1.59 (0.76–3.30)	1.39 (0.60–3.25)	1.05 (0.43–2.56)

Semi-adjusted model adjusted for age, gender, marital status, having children, commuting distance, employment duration, mean contract percentage, working overtime, and previous sickness absence days. Job classification variables special operational requirements, knowledge requirements, and aggravated working conditions were added to the fully adjusted model

Bold: *P* < 0.05

## Discussion

The results of this study showed that exposure to shift work or night shifts did not result in an increased risk for sickness absence when job classification variables were taken into account. Only the group of employees that changed into shift work that included night shifts did show an increased risk for long-term sickness absence (>7 days). Moreover, single employees or employees without children who worked in a three-shift system were at higher risk of long-term sickness absence compared to single employees or employees without children in day work. These employees, plus divorced and/or widowed employees, were also at higher risk for long-term sickness absence when they changed from one shift schedule to another.

The job classification system used in our study took into account both psychosocial and physical work factors [36]. In the review of Allebeck & Mastekaasa [20], moderate evidence was found for an effect of physically demanding work, and low psychological control both short- and long-term on sickness absence. More recent studies also found that high physical job demands and psychosocial risk factors increase sickness absence in various populations of employees [18, 22, 38, 39]. However, previous research has also shown that employees that work outside regular working hours can be more exposed to low job control and high physical demands compared to day workers [40], for which psychosocial and physical work factors might be considered as mediators in the association between shift work and sickness absence as well. Therefore, in order to obtain a precise estimate of the association between exposure to shift work and sickness absence, the multicausality of sickness absence has to be taken into account [17, 23, 29]. Our findings indicate that psychosocial and physical work factors, operationalized through job classification variables, are important to include as either confounders of mediating factors when analyzing this association.

It has been argued that there is inconclusive evidence for an association between rotating shift work and sickness absence [29]. In line with our findings, several studies did not find an association between rotating shift work and sickness absence [41, 42]. Other studies found higher sickness absence in rotating shift workers in three [43, 44], or two-shift [43, 45] schedules. However, Ohayon et al. [45] did not adjust for job characteristics, while Morikawa et al. [44] only took into account physical work demands. Niedhammer et al. [43] did adjust for psychological and physical workload in a large sample of French employees, and found that male employees involved in both two- and three-shift schedules had a higher risk of long-term sickness absence (>8 days). However, in all studies above, previous sickness absence of the studied population was not taken into account, although it has been shown that sickness absence in the past is a strong predictor for future sickness absence [21, 46], as was also shown in the present study (Table 2). Therefore, taking into account the previous sickness absence of the employees might explain the contrasting results of the present study as compared to others.

In addition, employees who were exposed to the same type of shift schedule during 2005 to 2008 may have learned to cope with their irregular schedules, implementing their working hours into their social and family life [47–49]. This lack of adaption might be one of the reasons for the finding that employees that changed into shift work that included night shifts showed an increased risk for long-term sickness absence. It is however also possible that these employees differed considerably from other shift workers in terms of lifestyle or household composition, that might have had an influence on sickness absence. Nevertheless, a post-hoc analysis showed that employees that started with night shifts were somewhat younger than other shift workers, but did not differ in terms of gender, household composition or commuting distance.

We found that shift work employees who had children did not have an increased risk for sickness absence, nor had shift work employees who were married or cohabiting. These findings may seem counterintuitive as non-standard working hours are generally thought to have a negative influence on social activities and work-family life balance, possibly leading to health problems and sickness absence [4, 50, 51]. Haines et al. [4], for instance, showed that work-family conflict partially mediates the relationship between shift work and health deterioration. Demourouti et al. [52], however, did not find an association between rotating shift work and work-family conflict, absenteeism and subjective health. The authors suggest that rotating shift work might increase opportunities for more common time with the family. Moreover, two recent studies showed that work-family conflict decreases if shift workers are able to choose their own shift schedule [53, 54]. Therefore, it might be possible that the married/cohabiting shift workers in our population have actively chosen to continue working in their rotating shift schedules.

Whereas common time with the family might counteract the negative effects of rotating shift work, this might not hold true for single or childless employees, as we found that these employees who worked in a three-shift schedule had a higher risk for long-term sickness absence, compared to singles or people without children in day work. In addition, single employees and employees without children also showed to have an increased risk for long-term sickness absence when they started to working in a three-shift schedule. An even larger relative risk was found for divorced or widowed employees who began working in a schedule that included night shifts (OR 3.96, 95%CI 1.24–12.69). A lack of family support, which can help employees to adapt to, and cope with a shift schedule, could contribute to sleeping problems and fatigue, possibly leading to medium- and long-term sickness absence [49, 50, 55],

Regarding the analyses of the number of sickness absence episodes, it was found that, compared to day workers, all employees involved in shift work had a lower risk for more sickness absence episodes. Frequent night shift exposure led to a lower risk for more sickness absence episodes as well. This lower incidence rate of sickness absence episodes among shift workers has been found before [56]. It is suggested that shift workers exhibit greater solidarity towards their shift colleagues and avoid being absent unexpectedly [52]. Furthermore, shift workers might regard, as opposed to day workers, non-severe complaints (e.g. not feeling well, fatigue, disturbed sleep) as a part of their work and not as reasons to call in sick [57].

The major strength of this study is the large historic cohort, with extensive, standard available company data of more than 7500 employees over a 5-year period. Because both work schedule and sickness absence data originated from company records, recall bias can be ruled out. The ground staff employees originated from a wide range of jobs throughout the company including both blue and white collar workers (e.g. gate agents, office employees, technical staff, baggage handlers), for which results can be generalizable for other companies as well.

We decided to analyse the association of shift work with sickness absence using an episode of more than 7 consecutive days because such an episode is an indication for medically certified sickness absence [15, 34, 58]. Still, a wide variation of sickness absence cut-offs have been used in other studies [from 1 day up to several weeks], for which results are not always comparable to our findings. The possibility exists that we would have found different results when sickness absence was operationalised differently [28, 42–45, 59]. However, because short-term sickness absence can be a good indication for coping strategies and the attitude towards work, we looked at the number of sickness absence episodes as well [20, 21, 25]. Our results showed that the association between shift work exposure and long-term sickness absence differed considerably from the association between shift work exposure and the number of sickness absence episodes. It is therefore important to include both short- and long-term sickness absence outcome measures when analysing the association between shift work and sickness absence.

Although we included numerous potential confounders to determine the association between shift work exposure and sickness absence. A part of the confounder information was based on a job classification system. Although this system is widely used to describe and evaluate functions in the Netherlands, there is no published information available about its validity. In addition, the job classification system is not able to take into account individual differences between jobs, for which it is possible we overadjusted our analyses. Overadjustment might also have occurred due to a systematic higher workload of the shift workers, which would have resulted in a systematic higher score on aggravated working conditions in the fully adjusted model. However, a beforehand conducted crosstab analyses showed that there was sufficient variation of aggravated working conditions scores within the shift type groups. We might also have overadjusted by using previous sickness absence as a confounder since this absent might have been due to the specific shift work exposure. On the other hand, adjusting for previous sickness absence does take into account the reduced exposure to a certain schedule between 2005 and 2008, in relation to the outcome measure that was analysed during the year 2009.

Previous research showed that health conditions, lifestyle, psychosocial determinants, and coping mechanisms can also play an important role in both the development of sickness absence [17, 20, 21, 23] and the tolerance to irregular working hours [49, 60]. Because we only used indirect, standard available company data, it was not possible to include all of these determinants in our analyses, which can have affected our results. Additionally, it was not possible to include lifestyle factors, such as smoking, alcohol or body weight, which might have confounded the association between shift work exposure and sickness absence. Fekeduleng et al. [61], for instance, showed that the increased sickness absence in night workers only held true for overweight employees, while no significant differences in sickness absence days between day, evening, and night shift workers was found for employees with a normal weight.

Next, it was decided to exclude the employees whose job was not evaluated by job classification system. Since these employees predominantly held an expert or high management function, the exclusion affected the external validity of the results. Finally, our results are probably biased due to selection effects. To make sure that the exposure period for all employees was equal, employees who were employed after 2005, and had incomplete data about working hour exposure in their first year of employment, were excluded from the analyses. Moreover, the MORE cohort did not include employees who terminated their employment before 2010, nor did we have any information about this group of employees. They might, however, have left the company due to health problems as a result of their (shift) work schedules. Consequently, only the employees who managed to keep working during the 5-year exposure period were analysed, introducing the healthy worker effect. Therefore, analysing the selected employees might have led to an underestimation of the association between shift work and sickness absence. Because of the fact that we did not have information about the employees before 2005 it is possible that the group of day workers included a substantial number of previous night workers. Because we could not exclude these employees, our results might have been diluted.

Our results show that it is important to take into account previous sickness absence and psychosocial and physical work factors to obtain a good estimation of the effect of shift work exposure on sickness absence. In our study population, exposure to different types of shift schedules was not associated with more long-term sickness absence, nor was cumulative night shift exposure. Instead, within the subgroups of single, childless or divorced employees, the risk for more long-term sickness absence did increase, when exposed to a three-shift schedule. Future research is needed to confirm our findings in other populations. It would for instance be possible to use similar cohorts, based on company data, for more sophisticated types of analyses, taking into account sickness absence changes over time. In addition, it should also be further explored what the influence of household composition and other possible mediating factors is.

Because it was found that employees that switched into a three-shift schedule showed an increases risk for long-term sickness absence, it seems useful to support employees who have to adapt to a new shift schedule. For this purpose, counselling and training at work through specific educational programs, involving sleep hygiene, nutrition, physical fitness, and exposure to light, to improve the coping mechanisms of the involved shift work employees, has been recommended [62, 63].

## Conclusions

Taking into account previous sickness absence and psychosocial and physical work factors, it was shown that continuous exposure to a two- or three-shift schedule did not result in an increased risk for sickness absence, neither did cumulative night shift exposure. Instead, shift work employees showed a lower risk for more sickness absence episodes compared to day workers. It was also found that employees who transferred into a shift schedule that included night shifts had an increased risk of future long-term sickness absence compared to day workers. Employees without children, single employees, and divorced or widowed employees did show a higher risk for long-term sickness absence when exposed or transferred to a three-shift schedule as well. Finally, it should be acknowledged that selection bias could not be ruled out and might have affected the results of this study.

## Abbreviations

CI: Confidence Interval
IRR: Incidence risk ratio
MORE: Monitoring Occupational Health Risks in Employees
OR: Odds Ratio
SD: Standard deviation
